# Neuroprotective Effects of Calmodulin Peptide 76-121aa: Disruption of Calmodulin Binding to Mutant Huntingtin

**DOI:** 10.1111/j.1750-3639.2008.00258.x

**Published:** 2010-01

**Authors:** Nichole L Dudek, Ying Dai, Nancy A Muma

**Affiliations:** 1 Department of Pharmacology and Experimental TherapeuticsMaywood, IL; 2 Neuroscience Program, Loyola University Chicago School of MedicineMaywood, IL; 3 Department of Pharmacology and Toxicology, University of Kansas School of PharmacyLawrence, KS

**Keywords:** adeno-associated viral vector, calcineurin, calmodulin, calmodulin-dependent kinase II, Huntington's disease, transglutaminase

## Abstract

Huntington's disease (HD) is a neurodegenerative disease caused by mutant huntingtin protein containing an expanded polyglutamine tract, which may cause abnormal protein–protein interactions such as increased association with calmodulin (CaM). We previously demonstrated in HEK293 cells that a peptide containing amino acids 76-121 of CaM (CaM-peptide) interrupted the interaction between CaM and mutant huntingtin, reduced mutant huntingtin-induced cytotoxicity and reduced transglutaminase (TG)-modified mutant huntingtin. We now report that adeno-associated virus (AAV)-mediated expression of CaM-peptide in differentiated neuroblastoma SH-SY5Y cells, stably expressing an N-terminal fragment of huntingtin containing 148 glutamine repeats, significantly decreases the amount of TG-modified huntingtin and attenuates cytotoxicity. Importantly, the effect of the CaM-peptide shows selectivity, such that total TG activity is not significantly altered by expression of CaM-peptide nor is the activity of another CaM-dependent enzyme, CaM kinase II. *In vitro*, recombinant exon 1 of huntingtin with 44 glutamines (htt-exon1-44Q) binds to CaM-agarose; the addition of 10 µM of CaM-peptide significantly decreases the interaction of htt-exon1-44Q and CaM but not the binding between CaM and calcineurin, another CaM-binding protein. These data support the hypothesis that CaM regulates TG-catalyzed modifications of mutant huntingtin and that specific and selective disruption of the CaM-huntingtin interaction is potentially a new target for therapeutic intervention in HD.

## INTRODUCTION

Huntington's disease (HD) is an autosomal dominant neurodegenerative disease characterized by chorea and cognitive disturbances (27). The gene involved in HD, interesting transcript 15 (IT15), encodes for the protein huntingtin (1). In HD, IT15 has an expanded CAG trinucleotide repeat in its first exon, resulting in a mutated form of the huntingtin protein containing an expanded polyglutamine tract in its N-terminus (34). Medium spiny neurons in the striatum are selectively vulnerable to neurodegeneration in HD (47). In HD brains, insoluble deposits that contain amino-terminal fragments of huntingtin with an expanded glutamine repeat are found in neurons (12). Huntingtin is a substrate of transglutaminase (TG), and, as the length of the glutamine repeat is increased, the protein becomes a better substrate (7, 16, 21, 23). It has been hypothesized that TG modifies huntingtin, thereby aiding in the stabilization of monomeric huntingtin (48), and the formation and stabilization of huntingtin containing aggregates (7, 17).

Huntingtin has been shown to interact with many proteins, including cyclic adenosine monophosphate (cAMP) response-element binding protein (CREB)-binding protein (CBP) (26), huntingtin-interacting protein 1 (HIP1), the spliceosome protein HYPA (18), and calmodulin (CaM) (3). It has also been reported that mutant huntingtin containing an expanded glutamine repeat interacts with CaM with a higher affinity than wild-type huntingtin (3). Interestingly, CaM, a calcium (Ca^2+^) binding protein that activates many enzymes, also interacts with TG and increases TG activity (36). We previously demonstrated that CaM colocalizes with TG2 and huntingtin protein in intranuclear inclusions in the HD cortex (49). Furthermore, inhibition of CaM results in decreased TG-catalyzed modifications of huntingtin in cells expressing mutant huntingtin and TG2 (49).

TGs (EC2.3.2.13) are a family of enzymes that catalyze a calcium-dependent acyl-transfer reaction between the γ-carboxy group of a protein-bound glutamine and either the ε-amino group of a protein-bound lysine or a primary amine, resulting in the formation of an ε-(γ-glutamyl) lysine bond (15). TG mRNA, protein levels and activity are all elevated in an HD brain (23, 25, 50). TG-catalyzed ε-(γ-glutamyl) lysine bonds and TG2 both colocalize with huntingtin in intranuclear inclusions in HD brain (48). Treatment of HD-transgenic mice with cystamine, a TG inhibitor, or knocking out TG2 increases survival (2, 11, 28, 46). Similarly, treating cells that express N-terminal mutant huntingtin and TG2 with cystamine increases cell survival and decreases the amount of TG-catalyzed modifications of huntingtin (50).

In HD, mutant huntingtin may alter the normal interaction between CaM and TG. One result could be an increased interaction between CaM and huntingtin resulting in a subsequent increase in TG activity. Another possible result could be sequestration of CaM, thereby inhibiting its biochemical functions such as activation of nitric oxide synthase (NOS), an enzyme which has been shown to have decreased activity in an HD mouse model (8). Previously, in HEK-293 cells transiently expressing N-terminal huntingtin with an expanded polyglutamine repeat and TG2, we found that a peptide containing amino acids 76–121 of CaM (CaM-peptide) and encoded by a fragment of exons 4 and 5 of the *CaM* gene was able to decrease the TG-catalyzed modifications of mutant huntingtin, the cytotoxicity induced by mutant huntingtin and the binding of mutant huntingtin to CaM (13). The goal of the current study was to examine the effect of adeno-associated viral (AAV) mediated-expression of that peptide (CaM-peptide) in differentiated neuroblastoma SH-SY5Y cells that stably express an N-terminal (63 amino acids in length) fragment of huntingtin containing 148 glutamines (SH-SY5Y-htt-N63-148Q cells). Previous studies demonstrated that a fragment of CaM from amino acid 78 to 148 was able to inhibit CaM-induced stimulation of phosphodiesterase and myosin light chain kinase (MLCK) (29, 30). Therefore, we hypothesized that CaM-peptide would compete with endogenous full-length CaM for binding to mutant huntingtin, resulting in inhibition of the endogenous CaM-mutant huntingtin interaction. We examined the effects that CaM-peptide had on TG-catalyzed modifications of mutant huntingtin, cytotoxicity associated with mutant huntingtin, total TG activity and binding of CaM to exon 1 of mutant huntingtin.

## MATERIALS AND METHODS

### AAV vector construction

A fragment of exons 4 and 5 of the *CaM* gene, encoding amino acids 76–121 of CaM [MKDTDSEEEIREAFRVFDKDGNGY ISAAELRHVMTNLGEKLTDEEV (CaM-peptide)], was cloned into pEF6. The following website, http://darwin.nmsu.edu/bioinfo/seqmake/seqmake.php, was used to randomly scramble the amino acid sequence of CaM-peptide: GDTVEREKDAYNSLEGFDNTIHTLRADIGMVEEVAKSKRDEEFLE (scram-CaM-peptide). This sequence was then analyzed at the National Center for Biotechnology Information (NCBI) site using the basic local alignment search tool (BLAST) network service to identify sequence similarity with the sequences of other proteins. Once it was determined that there was no significant similarity with any other protein sequence, the sequence was synthesized and cloned into pZERO-2 vector (IDT DNA Technologies, Coralville, IA, USA). The pEF6 vector encoding CaM-peptide and the pZERO-2 vector encoding scram-CaM-peptide were used as templates in a multi-step polymerase chain reaction (PCR) method. CaM-peptide or scram-CaM-peptide was amplified using primers 1 and 2 or 4 and 5, respectively, containing *XhoI* and *EcoRI* restriction sites. The resultant PCR products were digested and cloned into the *XhoI* and *EcoRI* sites in the pMig vector (generous gift from Dr Vinay Kumar, University of Chicago), which encodes for an internal ribosome entry site (IRES) followed by green fluorescent protein (GFP). The resultant CaM-peptide-IRES-GFP and scram-CaM-peptide-IRES-GFP pMig vectors were used as templates to amplify CaM-peptide-IRES-GFP and scram-CaM-peptide-IRES-GFP using primers 1 and 3, and 4 and 3, respectively, containing *XhoI* and *KpnI* restriction sites. The resultant PCR products were digested and cloned into the *XhoI* and *KpnI* sites in the pGAN vector (Gene Transfer Vector Core, University of Iowa, Iowa City, IA, USA) containing cytomegalovirus (CMV) promoter and bovine growth hormone polyadenylation signal. The resultant CMV-CaM-peptide-IRES-GFP and CMV-scram-CaM-peptide-IRES-GFP pGAN vectors were then subcloned into *NotI* restriction sites in the pFBGR vector (Gene Transfer Vector Core, University of Iowa, Iowa City, IA, USA) containing AAV elements. Constructs were verified by restriction digests and sequencing. The resultant vectors as well as an empty pFBGR vector, which encodes for GFP only, were then used for production of the AAV 2 expressing either CaM-peptide and GFP (AAV-CaM-peptide + GFP), scram-CaM-peptide and GFP (AAV-scram-CaM-peptide + GFP), or only GFP (AAV-GFP) (Gene Transfer Vector Core, University of Iowa, Iowa City, IA, USA).

PCR primers used for generating vectors:

- Primer 1: ACT CGA CTC GAG ATC ATG AAG GAC ACA GAC AGT GAG;
- Primer 2: ACT CGA GAA TTC CTA CAC CTC CTC ATC GGT CAG CTT C;
- Primer 3: ACT CGA GGT ACC TTA CTT GTA CAG CTC GTC CAT GCC;
- Primer 4: ACT CGA CTC GAG ATC ATG GGC GAT ACC GTG GAA CG;
- Primer 5: ACT CGA GAA TTC CTA TTC CAG AAA TTC TTC ATC ACG.

### Cell culture

Human SH-SY5Y cells were grown at 37°C in RPMI 1640 medium (Invitrogen, Carlsbad, CA, USA) containing 4 mM glutamine, 5% fetal bovine serum (FBS), 10% horse serum, 100 units/mL penicillin and 100 µg/mL streptomycin in the presence of 5% CO_2._ Cells were grown to 80%–90% confluency and transfected with a 63-amino acid N-terminal huntingtin fragment containing 148 polyglutamine repeats (htt-N63-148Q) using Lipofectamine Plus (Invitrogen, Carlsbald, CA, USA). Cells stably expressing htt-N63-148Q were selected based on their resistance to blasticidin. Cells were treated with 10 µM retinoic acid (Sigma, St. Louis, MO, USA) for 4 days to induce differentiation and upregulate TG expression (44), and then were infected with either AAV-CaM-peptide + GFP, AAV-scram-CaM-peptide + GFP or AAV-GFP at a multiplicity of infection (MOI) of 50.

### Fluorescence microscopy

Cells were infected with either AAV-CaM-peptide + GFP, AAV-scram-CaM-peptide + GFP or AAV-GFP, and plated onto collagen-coated chamber slides. Forty-eight hours post-infection, cells were washed in phosphate buffered saline (PBS) and fixed in 4% paraformaldehyde for 20 minutes at room temperature, then washed again in PBS. Coverslips were mounted with media containing 4′,6-diamidino-2-phenylindole (DAPI). Cells were examined using an Olympus (Tokyo, Japan) fluorescent inverted research microscope configured with Image-Pro Plus 4.5 software (VayTek, Fairfield, IA, USA). Monochrome images were captured using Retiga EX 1350 camera and Volume Scan 3.1 software (VayTek, Fairfield, IA, USA) and were pseudocolored and merged using Image-Pro Plus 4.5 software.

### Flow cytometry

Forty-eight hours post-viral infection, cells were harvested and centrifuged at 1500 g for 5 minutes. Cell pellets were washed twice in PBS with 1% bovine serum albumin (BSA). The cell pellets were then resuspended in 300 µL PBS with 1% BSA and were analyzed on a GFP channel using BD FACS™ caliber flow cytomer (BD Biosciences). Data were analyzed on the summit software suite (Dako Cytomation, Carpenteria, CA, USA).

### Immunoprecipitation

Forty-eight hours post-viral infection, cells were harvested and resuspended in lysis buffer containing 50 mM Tris–HCl, pH 8.8, 100 mM NaCl, 5 mM MgCl_2_, 0.5% NP-40, 1 mM ethylenediaminetetraacetic acid (EDTA) (19) and 1:1000 protease inhibitor mixture (Sigma, St. Louis, MO, USA). Insoluble fractions were prepared by centrifuging the cell lysates at 12 000 g for 5 minutes, removing the supernatant, resuspending the pellet in 95% formic acid and incubating at 37°C for 40 minutes. The formic acid was then removed under vacuum (19). The pellets were then resuspended in 10 mM Tris–HCl, pH 7.5, 0.14 M NaCl and 0.1% Tween 20, for further analysis. Immunopurification of proteins containing TG-catalyzed ε-(γ-glutamyl) lysine bonds was performed using 81D4 Mab pre-bound to sepharose beads using a protocol developed by CovalAb (Lyon, France) and as previously described (31). The eluted immunopurified proteins were stored at −80°C until immunoblot analysis.

### Immunoblots

Proteins were separated on 12% sodium dodecyl sulfate (SDS)-polyacrylamide gels then electrophoretically transferred to nitrocellulose membranes. Membranes were blocked in 5% nonfat dried milk in Tris-buffered saline (TBS) with 0.1% Tween 20. After overnight incubation in primary antibody, membranes were incubated in secondary antibody conjugated to horseradish peroxidase (Jackson ImmunoResearch, West Grove, PA, USA). Signal was detected using enhanced chemiluminescence (ECL) Western blotting detection reagents (Amersham Biosciences, Piscataway, NJ, USA). Immunoblots were quantified by calculating the sum of the densities of all of the pixels within each protein band as the integrated optical density (IOD) using Scion Image for Windows (Scion, Frederick, MD, USA). Film background IOD was measured and subtracted from the IOD of each band. Measurements were done in triplicate and the means were calculated.

### Antibodies

The mouse anti-myc monoclonal antibody directed against the amino acid sequence EQKLISEEDL was used at a 1:1000 for immunoblots (Invitrogen, Carlsbad, CA, USA) in order to detect N-terminal huntingtin. The mouse anti-actin antibody was used at 1:10 000 for immunoblots (MP Biomedicals, Solon, OH, USA) in order to detect actin.

### Cytotoxicity assay

Forty-eight hours post-viral infection, lactate dehydrogenase (LDH) assays were performed on the cells using the CytoTox 96® Non-Radioactive Cytotoxicity Assay (Promega, Madison, WI, USA). Briefly, half the media from the cells was removed and placed in the wells of a 96-well plate. Then, the cells were frozen in the remaining media at −80°C followed by thawing in order to lyse the cells. The cells and media were briefly centrifuged and the media was removed and placed in the remaining wells of the 96-well plate. Substrate solution was added to all samples and incubated at room temperature in the dark for 30 minutes. Stop solution was added, and the absorbance was recorded at 490 nm. Percent cytotoxicity was determined by the ratio of LDH activity in the media before lysing the cells to the LDH activity in the media after the cells were lysed.

### *Ex vivo* TG assay

In this *ex vivo* assay, TG activity is measured in cell lysates. 96-well Immulon 4 HBX plate (Dynatech, Franklin, MA, USA) was coated with N-N dimethylcasein (Sigma, St. Louis, MO, USA) in sodium bicarbonate overnight at 4°C. Then, the plates were washed with PBS and blocked with 2% milk in PBS for 1 h at 37°C, followed by three washes with PBS. Forty-eight hours post-viral infection, cells were harvested and lysed in 0.1 M Tris–HCl, pH 8.3, 1 mM EDTA, 1 mM phenylmethanesulfonyl fluoride (PMSF) and protease inhibitor cocktail. Cell lysates (25 µg of protein/sample) were diluted in 0.1 M Tris–HCl pH 8.5, 0.15 M NaCl, 5 mM dithiothreitol (DTT), 0.5 mM biotin labeled amine and 5 mM CaCl_2_. The mixture was then added to the wells in triplicate and incubated for 1 h at 37°C. The plate was washed with TBS and 0.001% Tween followed by incubation with streptavidin conjugated to HRP (Jackson ImmunoResearch, West Grove, PA, USA) for 1 h at room temperature. After a final wash, 1 × tetramethylbenzidine (TMB) substrate solution (eBioscience, SanDiego, CA, USA) was added and color was allowed to develop for 5–10 minutes. Then, the reaction was stopped with 3 N sulfuric acid and the absorbance was read at 450 nm.

### *In situ* TG assay

In this *in situ* assay, TG activity is measured in intact cells via incorporation of a polyamine and the presence of the TG-catalyzed bond. Forty-two hours post-viral infection, cells were treated with 2 mM 5-(biotinamido)pentylamine, a biotinylated polyamine (Pierce, Rockford, IL, USA), and 6 h post-polyamine treatment, cells were harvested and lysed in 0.1 M Tris–HCl pH 8.3, 1 mM EDTA, 1 mM PMSF and protease inhibitor cocktail. 96-well Immulon 4 HBX plates were coated with anti-ε-(γ-glutamyl) lysine (81D4) antibody (CovalAb, Lyon, France) in 0.1 M Na_2_HPO_4_·7 H_2_O pH 9 overnight at 4°C. Then the plates were washed with PBS and blocked with 2% milk in PBS for 1 h at room temperature, followed by 3 washes with PBS with 0.0375% Tween. Cell lysates (50 µg of protein/sample/well) were added to the wells in triplicates and incubated for 1 h at 37°C. The plate was washed with PBS and 0.0375% Tween, followed by incubation with streptavidin conjugated to HRP (Jackson ImmunoResearch, West Grove, PA, USA) for 1 h at room temperature. After final washes, a 1 × TMB substrate solution was added and color was allowed to develop for 5–10 minutes. The reaction was stopped with 3 N sulfuric acid and the absorbance was read at 450 nm.

### CaM kinase II activity assay

CaM kinase II enzyme activity was analyzed using the SignaTECT® Calcium/Calmodulin-Dependent Protein Kinase Assay System (ProMEGA, Madison, WI, USA), according to the manufacturer's protocol as well as without the addition of exogenous CaM. Briefly, SH-SY5Y cells were transfected with vector (V), htt-N63-148Q (H) + V, CaM-peptide + V, or H + CaM-peptide using Lipofectamine Plus (Invitrogen, Carlsbald, CA, USA). Forty-eight hours post-transfection cells were lysed in CaM kinase extraction buffer [20 mM Tris–HCl, pH 8.0, 2 mM EDTA, 2 mM ethylene glycol tetraacetic acid (EGTA), 2 mM DTT, 1 mM PMSF and 1:1000 protease inhibitor cocktail (Sigma, St Louis, MO, USA)]. Cell lysates were mixed with (γ-^32^P) adenosine tri-phosphate (ATP) (at 3000 Ci/mmol, 10 mCi/mL), activation buffer containing 5 mM CaCl_2_ and 5 µM calmodulin or control buffer containing 5 mM EGTA, with and without biotinylated CaM kinase II substrate. After incubation at 30°C for 2 minutes, the reaction was terminated by adding 7.5 M guanidine hydrochloride and spotted to a streptavidin-impregnated membrane. Membranes were washed and retained radioactivity was quantitated by liquid scintillation counting (Beckman, Fullerton, CA, USA). Radioactive counts were converted to endogenous CaM kinase II activity in the sample by the following formula:


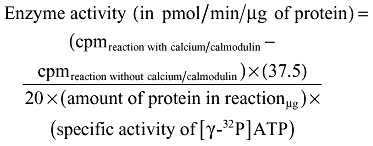


### Expression and purification of recombinant mutant huntingtin protein

*Escherichia coli* expressing a modified pMAL vector (New England Biolabs, Ipswich, MA, USA) encoding exon 1 of huntingtin with 44 glutamine residues (kindly provided by Dr Christopher Ross, The Johns Hopkins University School of Medicine, Baltimore, MD) were grown to an OD_600_ of 0.6–0.8, induced by adding 0.3 mM isopropyl-β-D-thiogalactopyranoside followed by shaking for 2 h at 28°C, and then centrifuged for 15min at 5000 g. The cell pellets were then resuspended in lysis buffer [PBS pH 7.4, supplemented with 10 mM methionine, 2 mM EDTA, 5 mM dithiothreitol, 1 mM 4-(2-aminoethyl)benzenesulfonyl fluoride, and protease inhibitor mixture (Sigma, St. Louis, MO)] and samples were frozen in a dry ice-ethanol mixture. Samples were then thawed on ice followed by sonication and then centrifuged at 20 000 g for 15 minutes. To purify maltose binding protein (MBP) fusion proteins, the supernatant was applied to an amylose resin (New England Biolabs, Ipswich, MA, USA) packed column (2.5 × 10 cm) that had first been washed with eight column volumes of PBS pH 7.4. After addition of the lysate supernatant, the column was then washed with 12 column volumes of PBS pH 7.4, followed by elution of the fusion protein with two column volumes of PBS with 10 mM maltose. The fusion protein was further purified by incubating with Ni-NTA agarose (Qiagen, Valencia, CA, USA) in PBS supplemented with 10 mM imidazole with rocking for 2 h at 4°C. The resin was then washed three times with PBS supplemented with 20 mM imidazole, and fusion protein was eluted with six resin volumes of PBS supplemented with 250 mM imidazole. Purified fusion protein was analyzed by SDS-polyacrylamide gel electrophoresis (PAGE) and visualized by Coomassie staining.

### *In vitro* mutant huntingtin-CaM binding

Binding of purified mutant huntingtin to calmodulin was studied using CaM-agarose (Sigma, St. Louis, MO, USA). Purified mutant huntingtin protein (8.4 µg/mL) or purified calcineurin protein (1.4 µg/mL, positive control; Sigma, St. Louis, MO, USA), with or without varying concentrations of purified CaM-overlap peptide (Biopeptide Co. Inc., San Diego, CA, USA) and/or varying concentrations of N-(6-Aminohexyl)-1-naphthalenesulfonamide hydrochloride (W-5), a calmodulin antagonist (Tocris, Ellisville, MO, USA), were mixed with 45 µg/mL CaM-agarose in 10 mM Tris–HCl (pH 8) containing 1 mM CaCl_2_ and 150 mM NaCl, and then incubated overnight at 4°C. After washing three times with 20 mM Hepes pH 7.4, 150 mM NaCl, 5 mM sodium pyrophosphate, 10% glycerol, 1% Triton X-100, 1 mM CaCl_2_ and protease inhibitor mixture (Sigma, St. Louis, MO, USA), and once with 10 mM Hepes pH 7.4, the agarose-bound proteins were eluted by incubation at 100°C for 10 minutes in Laemmli sample loading buffer. The fractions obtained were analyzed by SDS-gel electrophoresis followed by immunoblotting.

### Statistics

All statistical analyses were performed using GB Stat software (Dynamic Microsystems, Inc., Silver Spring, MD, USA). Data are expressed as means ± standard error of the mean (SEM). Analysis of Variance (ANOVA) or a two-tailed Student's *t*-test was used to analyze the data. Post-hoc comparisons were conducted using a Newman–Keuls test.

## RESULTS

### AAV-mediated expression of CaM-peptide in human neuroblastoma SH-SY5Y cells that stably express N-terminus of mutant huntingtin

**Figure 1:**
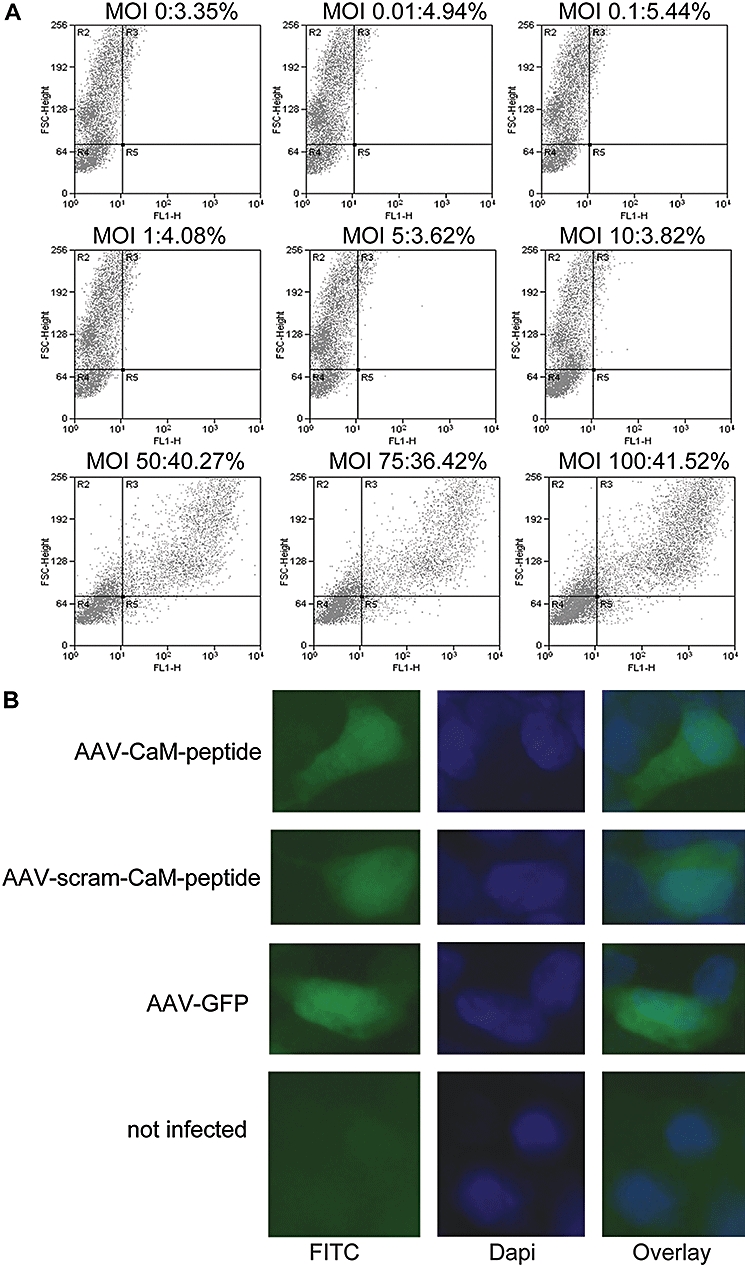
*AAV-mediated expression of GFP in SH-SY5Y cells*. **A.** SH-SY5Y cells were infected with varying MOIs (0, 0.01, 0.1, 1, 5, 10, 50, 75, 100) of AAV-GFP. Forty-eight hours post-infection cells were assessed for percent AAV-mediated GFP expression by flow cytometry. **B.** Differentiated SH-SY5Y were infected with either AAV-CaM-peptide, AAV-scram-CaM-peptide or AAV-GFP (MOI = 50). Forty-eight hours post-infection cells were examined with fluorescence microscopy to examine AAV-mediated GFP expression. Abbreviations: AAV = adeno-associated virus; CaM = calmodulin; FITC = fluorescein isothiocyanate; GFP = green fluorescent protein; MOI = multiplicity of infection.

We developed three different AAVs: an AAV which mediates expression of a peptide consisting of amino acids 76–121 of CaM, CaM-peptide, along with GFP (AAV-CaM-peptide + GFP), an AAV which mediates expression of a scrambled version of CaM-peptide along with GFP (AAV-scram-CaM-peptide + GFP) and an AAV which mediates expression of only GFP (AAV-GFP). In order to determine an appropriate MOI for the AAV, SH-SY5Y cells were transduced with varying MOIs (0, 0.01, 0.1, 1, 5, 10, 50, 75, 100) of AAV-GFP. Forty-eight hours post-infection cells were assessed for AAV-mediated GFP expression using flow cytometry (Figure 1A). An MOI of 50 resulted in the largest percentage of GFP-positive SH-SY5Y cells with the lowest level of cell death. To determine if an MOI of 50 resulted in similar intracellular distribution and expression of GFP in differentiated SH-SY5Y cells for all three AAV developed, we performed fluorescence microscopy. An MOI of 50 for each of the viruses showed similar AAV-mediated GFP expression in differentiated SH-SY5Y cells (Figure 1B).

**Figure 2:**
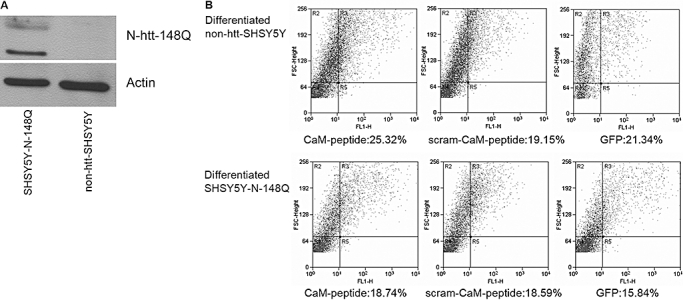
*AAV infection (MOI 50) of differentiated neuroblastoma SH-SY5Y cells stably expressing N-terminal mutant huntingtin*. **A.** Western blot analysis of human neuroblastoma SH-SY5Y cells stably expressing N-terminal mutant huntingtin. SH-SY5Y cells were transfected with N-terminal mutant huntingtin with an expanded polyglutamine repeat (SHSY5Y-htt-N63-148Q cells) and were selected based on their resistance to blasticidin. Blot was probed for N-terminal mutant huntingtin (upper) and actin (lower). **B.** Non-htt-SHSY5Y cells and SHSY5Y-htt-N63-148Q cells were treated with 10 µM retinoic acid for 4 days and then infected with either AAV-CaM-peptide, AAV-scram-CaM-peptide, or AAV-GFP (MOI = 50). Forty-eight hours post-infection cells were assessed for percent AAV-mediated GFP expression by flow cytometry. Abbreviations: AAV = adeno-associated virus; CaM = calmodulin; GFP = green fluorescent protein; non-htt-148Q = SH-SY5Y cells not expressing mutant huntingtin.

Next, we developed human neuroblastoma SH-SY5Y cell lines that stably express N-terminal mutant huntingtin. Expression levels of each cell line were estimated by Western blot analysis, and cell lines that showed moderate and similar expression were selected (Figure 2A). To further investigate the viral infection procedure, we used flow cytometry to quantitatively determine if an MOI of 50 would result in similar levels of infection for all three AAVs (AAV-CaM-peptide + GFP, AAV-scram-CaM-peptide + GFP or AAV-GFP) in differentiated SH-SY5Y cells not expressing mutant huntingtin (non-htt-SHSY5Y cells) and in differentiated SH-SY5Y cells that stably express mutant huntingtin (SHSY5Y-htt-N63-148Q cells). Non-htt-SHSY5Y and SHSY5Y-htt-N63-148Q cells were treated with 10 µM retinoic acid for 4 days to induce differentiation. Then, cells were infected with either AAV-CaM-peptide + GFP, AAV-scram-CaM-peptide + GFP or AAV-GFP (MOI = 50). Forty-eight hours post-infection, cells were assessed for GFP expression by flow cytometry. All three viruses infected differentiated non-htt-SHSY5Y and SHSY5Y-htt-N63-148Q cells with similar efficiencies (Figure 2B). However, these values were lower than previously measured for this MOI, perhaps caused by conditions associated with neuronal differentiation.

### AAV-mediated expression of CaM-peptide decreases TG-catalyzed modifications of mutant huntingtin in SH-SY5Y-htt-N63-148Q cells

**Figure 3:**
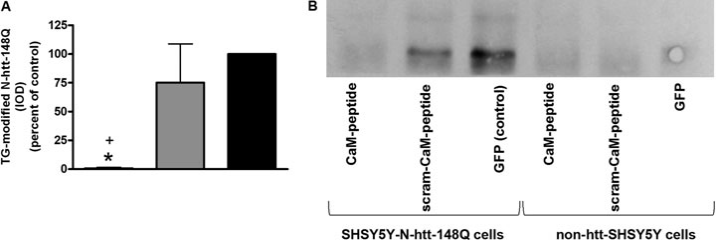
*The level of TG-modified N-terminal mutant huntingtin in the SHSY5Y-htt-N63-148Q cells expressing CaM-peptide was significantly lower than in SHSY5Y-htt-N63-148Q cells expressing GFP (control) or scram-CaM-peptide*. **A.** Quantification of immunoblot depicted graphically. **B.** Representative immunoblot of protein immunoprecipitated using the 81D4 antibody (for TG-catalyzed bonds) conjugated to sepharose. Blot was probed for N-terminal mutant huntingtin. Data shown are the mean IOD ± SEM (n = 3). One-way analysis of variance (F_(2,8)_ = 7.032, *P* < 0.0267) and Newman-Keuls comparison. * indicates *P* < 0.05 compared with SHSY5Y-htt-N63-148Q infected with AAV-GFP and + indicates *P* < 0.05 compared with SHSY5Y-htt-N63-148Q infected with AAV-scram-CaM-peptide. Abbreviations: AAV = adeno-associated virus; CaM = calmodulin; GFP = green fluorescent protein; IOD = integrated optical density; non-htt-148Q = SH-SY5Y cells not expressing mutant huntingtin; TG = transglutaminase.

With the cell and viral system now defined, we determined the effect of viral-mediated expression of CaM-peptide in differentiated SHSY5Y-htt-N63-148Q cells on TG-catalyzed modifications of N-terminal mutant huntingtin. Cells were differentiated, infected with either AAV-CaM-peptide + GFP, AAV-scram-CaM-peptide + GFP or AAV-GFP (MOI = 50), and forty-eight hours post-infection cells were assayed for TG-modified N-terminal mutant huntingtin. SHSY5Y-htt-N63-148Q cells expressing CaM-peptide had a significantly lower amount of TG-modified N-terminal huntingtin compared with cells expressing scram-CaM-peptide or only GFP (Figure 3).

### AAV-mediated expression of CaM-peptide decreases mutant huntingtin associated cytotoxicity in neuroblastoma cell lines that express N-terminal mutant huntingtin

**Figure 4:**
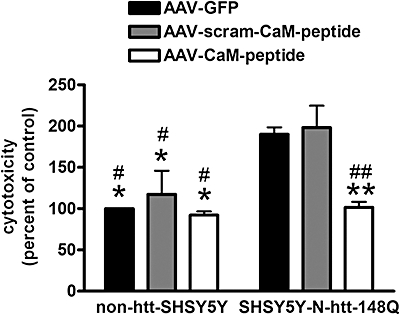
The level of cytotoxicity in SHSY5Y-htt-N63-148Q cells expressing CaM-peptide was significantly lower than in SHSY5Y-htt-N63-148Q cells expressing GFP (control) or scram-CaM-peptide. Lactate dehydrogenase levels were measured and used as an index of cytotoxicity. Data shown are the mean ± standard error of the mean (n = 3). Two-way ANOVA [Factor: Virus (F_(2,12)_ = 7.514, *P* = 0.0077)]; Factor: Huntingtin (F_(1,12)_ = 19.73, *P* < 0.0008.); Virus × Huntingtin (F_(2,12)_ = 3.552, *P* = 0.0614)) and Newman-Keuls comparison. * indicates *P* < 0.05 and ** indicates *P* < 0.01 compared with SHSY5Y-htt-N63-148Q cells infected with AAV-GFP. # indicates *P* < 0.05 and ## indicates *P* < 0.01 compared with SHSY5Y-htt-N63-148Q cells infected with AAV-scram-CaM-peptide. Abbreviations: AAV = adeno-associated virus; CaM = calmodulin; GFP = green fluorescent protein; TG = transglutaminase.

Differentiated non-htt-SHSY5Y or SHSY5Y-htt-N63-148Q cells were infected with either AAV-CaM-peptide + GFP, AAV-scram-CaM-peptide + GFP or AAV-GFP (MOI = 50), and forty-eight hours post-infection levels of cytotoxicity were measured. SHSY5Y-htt-N63-148Q cells expressing CaM-peptide had approximately half the level of cytotoxicity compared with SHSY5Y-htt-N63-148Q cells expressing scram-CaM-peptide or only GFP. Furthermore, there was no significant difference in the level of cytotoxicity between SHSY5Y-htt-N63-148Q cells expressing CaM-peptide and non-htt-SHSY5Y cells (Figure 4).

Differentiated SHSY5Y cells transiently transfected with either htt-N63-18Q or htt-N63-148Q were infected with either AAV-CaM-peptide + GFP or AAV-scram-CaM-peptide + GFP, or were uninfected (MOI = 50), and 96 h post-infection levels of cytotoxicity were measured using the LDH assay. In cells transiently transfected with htt-N63-18Q, we found that infection with AAV-CaM-peptide had no effect on cytotoxicity (38.1 ± 1.1; mean percent cytotoxicity ± SEM) compared with cells infected with AAV-scram-CaM-peptide (35.4 ± 2.4) or uninfected cells (38.0 ± 1.3). However, in these transiently transfected cells, there was also no increase in cytotoxicity in cells transfected with htt-N63-148Q (34.6 ± 2.3) compared with cells transfected with htt-N63-18Q (38.0 ± 1.3). The lack of toxicity of the htt-N63-148Q was perhaps caused by the lower levels of expression of the htt-N63 constructs in transiently transfected cells compared with stable transfections.

### Effect of AAV-mediated expression of CaM-peptide on total TG activity in neuroblastoma cell lines that stably express N-terminal mutant huntingtin

**Figure 5:**
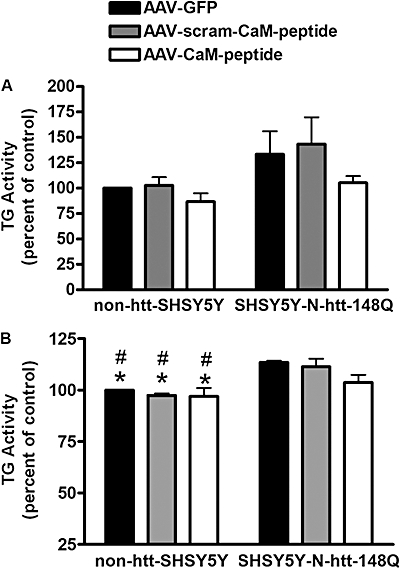
*There were no significant differences in total ex vivo or* in situ *TG activity in non-htt-SHSY5Y cells or SHSY5Y-htt-N63-148Q cells expressing CaM-peptide, scram-CaM-peptide or GFP*. **A.** There were no significant differences in total *ex vivo* TG activity in non-htt-SHSY5Y cells expressing either CaM-peptide, scram-CaM-peptide or GFP (control). There was a small but insignificant increase in total *ex vivo* TG activity in SHSY5Y-htt-N63-148Q cells expressing scram-CaM-peptide or GFP compared with non-htt-SHSY5Y cells and SHSY5Y-htt-N63-148Q cells expressing CaM-peptide. **B.** Similarly there were no significant differences in total *in situ* TG activity in non-htt-SHSY5Y cells expressing CaM-peptide, scram-CaM-peptide or GFP. However, total *in situ* TG activity was significantly less in non-htt-SHSY5Y cells compared with SHSY5Y-N63-htt-148Q cells expressing scram-CaM-peptide or GFP. But, total *in situ* TG activity in non-htt-SH-SY5Y cells was not significantly different from total TG activity in SH-SY5Y-htt-N63-148Q cells expressing the CaM-peptide. **A.** Data shown are the mean ± standard error of the mean (SEM; n = 3). Two-way analysis of variance (ANOVA) [Factor: Virus (F_(2,12)_ = 2.613, *P* = 0.1143)]; Factor: Huntingtin (F_(1,12)_ = 24.16, *P* < 0.0004); Virus × Huntingtin [F_(2,12)_ = 1.016, *P* = 0.3911] and Newman–Keuls comparison. **B.** Data shown are the mean ± SEM (n = 3). Two-way ANOVA [Factor: Virus (F_(2,12)_ = 1.722, p = 0.22)]; Factor: Huntingtin (F_(1,12)_ = 6.139, *P* < 0.0291); Virus × Huntingtin (F_(2,12)_ = 0.2775, *P* = 0.7624) and Newman–Keuls comparison. * indicates *P* < 0.05 compared with SHSY5Y-htt-N63-148Q cells infected with AAV-GFP. # indicates *P* < 0.05 compared with SHSY5Y-htt-N63-148Q cells infected with AAV-scram-CaM-peptide. Abbreviations: AAV = adeno-associated virus; CaM = calmodulin; GFP = green fluorescent protein; TG = transglutaminase.

Next, we examined the effect of expression of the CaM-peptide on total TG activity in differentiated non-htt-SHSY5Y and SHSY5Y-htt-N63-148Q cells. Cells were infected with either AAV-CaM-peptide + GFP, AAV-scram-CaM-peptide + GFP or AAV-GFP (MOI = 50), and harvested forty-eight hours post-infection. TG activity was measured *ex vivo* based on the incorporation of 5-(biotinamido)pentylamine into the N,N′-dimethylcasein substrate coated onto micro-plates. There were no significant differences in total TG activity between the different infections [AAV-CaM-peptide + GFP, AAV-scram-CaM-peptide + GFP or AAV-GFP (control)] in either non-htt-SHSY5Y cells or SHSY5Y-htt-N63-148Q cells (Figure 5A). There were small but insignificant increases in total TG activity in SHSY5Y-htt-N63-148Q cells expressing scram-CaM-peptide or only GFP, compared with non-htt-SHSY5Y cells expressing either GFP only, scram-CaM-peptide or CaM-peptide (Figure 5A). To determine whether cell lysis had an effect on enzyme activity, we measured total TG activity *in situ*. Non-htt-SHSY5Y and SHSY5Y-htt-N63-148Q cells were infected with one of the various AAV, and 42 h post-infection, were treated with 5-(biotinamido)pentylamine. Six hours later, cells were harvested and the cell lysates were applied to micro-plates coated with anti-ε-(γ-glutamyl) lysine (81D4) antibody in order to capture proteins that were modified by TG *in situ*. TG-catalyzed incorporation of 5-(biotinamido)pentylamine into cellular proteins was then determined by incubation with streptavidin-HRP. Similar to the *ex vivo* assay, there was no significant difference in total TG activity between the various infections [CaM-peptide, scram-CaM-peptide or GFP (control)] in SHSY5Y-htt-N63-148Q cells (Figure 5B). Similarly, there also was no difference in total TG activity in the various infections in non-htt-SHSY5Y cells. However, there was a significant decrease in total TG activity in non-htt-SHSY5Y cells compared with SHSY5Y-htt-N63-148Q cells expressing scram-CaM-peptide or only GFP. Total TG activity in non-htt-SHSY5Y cells was not significantly different from total TG activity in SHSY5Y-htt-N63-148Q cells expressing CaM-peptide (Figure 5B).

### CaM-peptide does not significantly affect CaM kinase II activity

**Figure 6:**
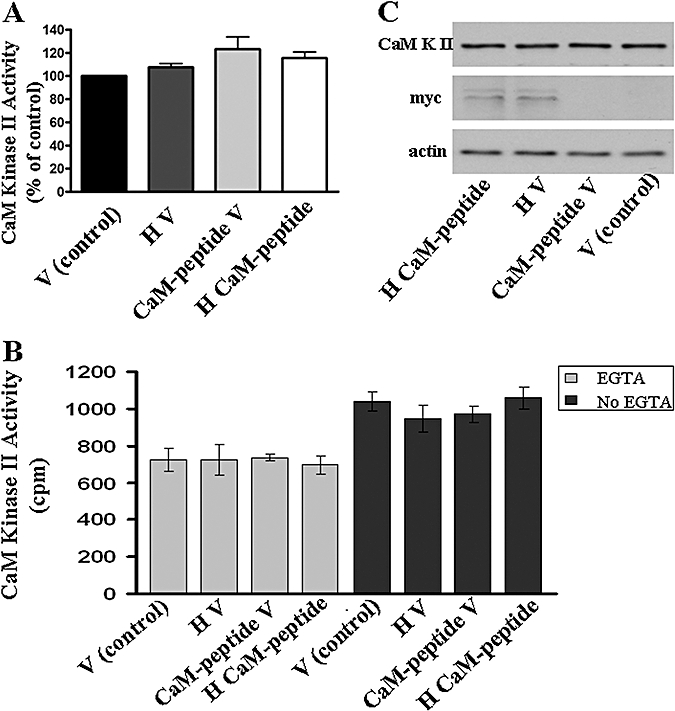
*Expression of CaM-peptide in N-terminal mutant huntingtin expressing cells does not significantly affect CaM kinase II activity or expression*. **A.** SH-SY5Y cells were transfected with vector (V), htt-N63-148Q (H) + V, CaM-peptide + V, or H + CaM-peptide. Cells were harvested 48 h post-transfection and CaM kinase II activity was evaluated by the SigmaTECT CaM kinase II Assay System as per the manufacturer's directions. Data shown are the mean ± standard error of the mean (SEM; n = 3). One-way analysis of variance (ANOVA) (F_(3,8)_ = 2.669, *P* = 0.1187) and Newman-Keuls Multiple comparison indicate no significant differences among groups. **B.** The CaM kinase II assay was also performed in the absence of exogenous calmodulin. Data shown are the mean ± SEM. Two-way ANOVA indicates a significant main effect of EGTA (*F*_(1,16)_ = 143.37, *P* < 0.0001). However, there was no significant main effect of transfection on CaM kinase II activity (F_(3,16)_ = 0.86, *P* = 0.48). The interaction between EGTA and transfection was also not significant (F_(3,16)_ = 1.89, *P* = 0.17). Newman-Keuls multiple comparison test indicates no significant differences in CaM kinase II activity among the various transfections in either the presence or absence of EGTA. **C.***Top*, protein expression of CaM kinase II remained at the same level after transfections of either htt-N-148Q (H), CaM-peptide or the combination of those two constructs. *Middle*, myc antibody was used to confirm the expression of htt-N-148Q. *Bottom*, equal loading was verified by reprobing the same membrane with an actin antibody. Abbreviations: CaM = calmodulin; EGTA = ethylene glycol tetraacetic acid; H = htt-N63-148Q.

To determine if expression of CaM-peptide has effects on the activity of other CaM-dependent enzymes, we examined whether expression of CaM-peptide affects CaM-dependent protein kinase II (CaM kinase II) activity. SH-SY5Y cells were transfected with vector, htt-N63-148Q, CaM-peptide or a combination of htt-N63-148Q and CaM-peptide. We found no significant differences in CaM kinase II activity among the various transfections (Figure 6). Interestingly, expression of CaM-peptide alone resulted in a small but insignificant increase in CaM kinase II activity compared with all other transfections. We also examined the activity of CaM kinase II without the addition of exogenous CaM (addition of exogenous CaM is recommended, as described in the manufacturer's protocol). There was no significant effect of CaM-peptide under these assay conditions either (Figure 6).

### CaM-peptide inhibits binding of N-terminal mutant huntingtin with an expanded polyglutamine repeat and CaM

**Figure 7:**
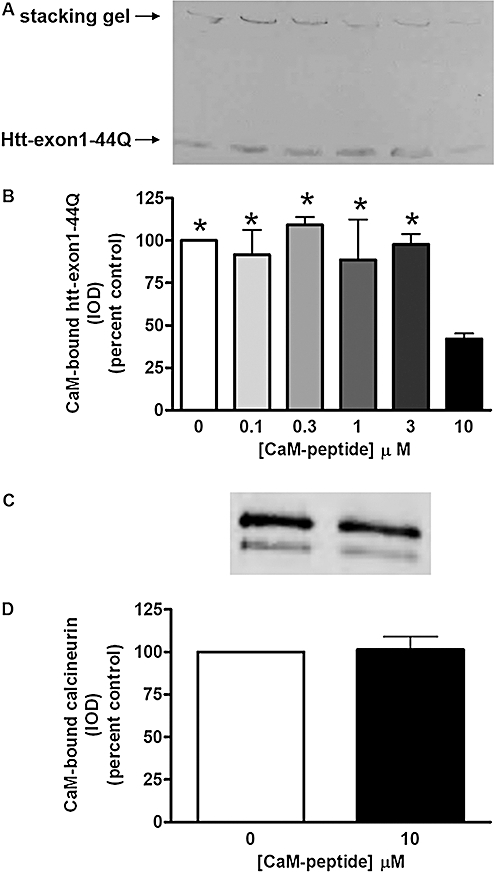
The level of CaM-bound huntingtin-exon 1 with an expanded polyglutamine repeat (htt-exon1-44Q) in the absence and presence of varying concentrations of CaM-peptide. The amount of CaM-bound htt-exon1-44Q was significantly lower when 10 µM of CaM-peptide was present than when no CaM peptide was present (control). Furthermore, lower concentrations of CaM peptide are required to inhibit self-aggregated htt-exon1-44Q. However, 10 µM of CaM-peptide did not affect the binding of CaM with calcineurin, a known CaM-binding protein. (**A**) Representative western blot of protein immunoprecipitated using CaM-agarose. Blot was probed for N-terminal mutant huntingtin. (**C**) Representative western blot of protein immunoprecipitated using CaM-agarose. Blot was probed for calcineurin. (**B** and **D**) Quantification of immunoblots depicted graphically. Data shown are the mean IOD ± standard error of the mean (n = 3). CaM-bound htt-exon1-44Q data: one-way analysis of variance (F_(5,17)_ = 3.931, *P* < 0.024) and Newman–Keuls comparison, * indicates *P* < 0.05 compared with 10 µM (CaM-peptide). CaM-bound calcineurin data: two-tailed *t*-test (*P* = 0.8575). Abbreviations: CaM = calmodulin; htt-exon1-148Q = recombinant exon 1 of huntingtin with 44 glutamines.

**Figure 8:**
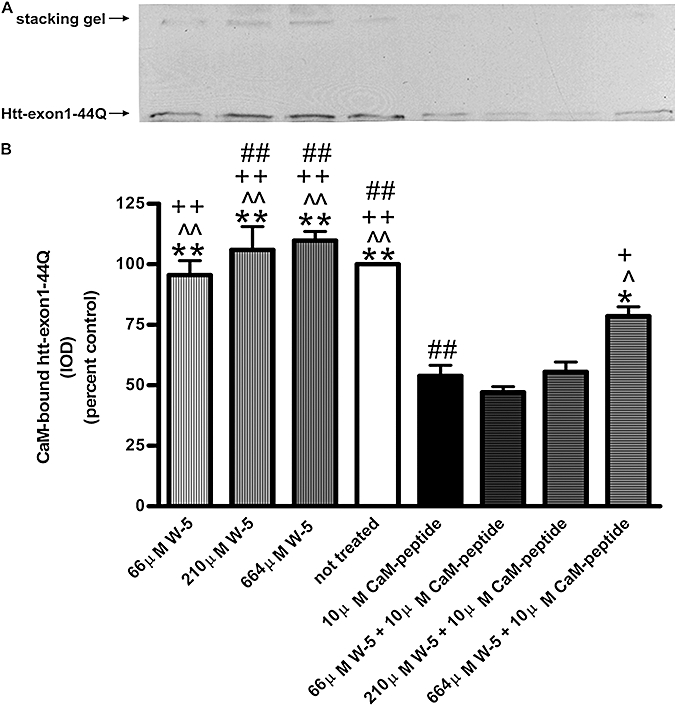
*The level of CaM-bound huntingtin-exon 1 with an expanded polyglutamine repeat (htt-exon1-44Q) in the absence and presence of varying concentrations of CaM-peptide and/or W-5*. **A.** The presence of W-5 did not alter the amount of CaM-bound htt-exon1-44Q. The amount of CaM-bound htt-exon1-44Q was significantly lower when 10 µM of CaM-peptide was present than when W-5 or no CaM peptide was present (control). However, the amount of CaM-bound htt-exon1-44Q was significantly increased when 664 µM W-5 is present along with 10 µM of CaM-peptide. (**A**) Representative western blot of protein immunoprecipitated using CaM-agarose. Blot was probed for N-terminal mutant huntingtin. **B.** Quantification of immunoblot depicted graphically. Data shown are the mean ± SEM (n = 3). Two-way ANOVA [Factor: W-5 (F_(3,23)_ = 7.712, *P* = 0.0021)]; Factor: CaM-peptide (F_(1,23)_ = 157.734, *P* < 0.0001); W-5 × CaM-peptide (F_(3,23)_ = 1.544, *P* = 0.2417) and Newman-Keuls comparison. ** indicates *P* < 0.01 compared with 10 mM (CaM-peptide). * indicates *P* < 0.05 compared with 10 mM (CaM-peptide). ^∧^ indicates *P* < 0.01 compared with 66 mM W-5 + 10 mM (CaM-peptide). ++ indicates *P* < 0.01 compared with 210 mM + W-5 10 mM (CaM-peptide). + indicates *P* < 0.05 compared with 210 mM W-5 + 10 mM (CaM-peptide). ## indicates *P* < 0.01 compared with 664 mM W-5 + 10 mM (CaM-peptide). # indicates *P* < 0.05 compared with 664 mM W-5 + 10 mM (CaM-peptide). Abbreviations: CaM = calmodulin; htt-exon1-44Q = recombinant exon 1 of huntingtin with 44 glutamines.

Thus far, all the experiments performed to examine the effects of CaM-peptide were done in cells where other endogenous proteins could play a role in mediating the action of CaM-peptide. Therefore, an *in vitro* assay was used to determine if CaM-peptide could directly interfere with the interaction of N-terminal mutant huntingtin and CaM. CaM-agarose was used to immunoprecipitate recombinant purified huntingtin exon 1 with an expanded polyglutamine repeat (htt-exon1-44Q) in the absence and presence of varying concentrations of CaM-peptide. The amount of CaM-bound htt-exon1-44Q was significantly lower when 10 µM of CaM-peptide was present than when CaM-peptide was absent (Figure 7A,B). To determine if CaM-peptide would non-specifically inhibit the interaction of CaM with any CaM-binding protein, we incubated calcineurin with CaM-agarose in the absence and presence of 10 µM of CaM-peptide. The presence of 10 µM of CaM-peptide did not affect the binding of CaM with calcineurin (Figure 7C,D). Next, to investigate the potential site of interaction of CaM-peptide, the CaM antagonist, W-5, was used along with CaM-peptide. W-5 alone did not affect the amount of CaM-bound htt-exon1-44Q, but once again the amount of CaM-bound htt-exon1-44Q was significantly lower when 10 µM of CaM-peptide was present. However, the amount of CaM-bound htt-exon1-44Q was significantly increased when 664 µM W-5 is present along with 10 µM of CaM-peptide (Figure 8).

## DISCUSSION

The disease-causing mutation in HD is an expanded polyglutamine repeat in huntingtin protein. This mutant form of huntingtin becomes a better substrate for TG (6, 16, 21, 23, 50) and has increased interaction with CaM (3, 49). Interestingly, TG is positively regulated by CaM (5, 36, 49). Therefore, we hypothesized that the interactions between mutant huntingtin, CaM and TG may be deleterious. Previously, we found in HEK-293 cells transiently expressing N-terminal mutant huntingtin, TG2 and a peptide containing amino acids 76–121 of CaM CaM-peptide that TG-catalyzed modifications of mutant huntingtin were reduced, cytotoxicity associated with mutant huntingtin protein was reduced and carbacol-stimulated calcium release was normalized (13). In order to test the effects of CaM-peptide in an HD transgenic mouse model, we created an AAV which mediates the expression of CaM-peptide. Before proceeding to an HD animal model, we first tested the effects of AAV-mediated expression of CaM-peptide in a neuronal HD cell model as neurons are affected in HD and the effects of CaM-peptide have only been tested in kidney cells. We created neuroblastoma SH-SY5Y cells which stably express N-terminal mutant huntingtin and endogenously express TG 2 (SHSY5Y-htt-N63-148-Q cells; Figure 2A) and examined the effect of AAV-mediated expression of CaM-peptide on TG-activity and mutant huntingtin-associated neurotoxicity. Furthermore, we induced differentiation of SHSY5Y cells prior to testing the effects of the CaM peptide to increase the levels of TG expressed in the cells and to acquire a neuronal phenotype, both effects producing a model more closely resembling cells that degenerate in HD.

AAV-mediated expression of CaM-peptide was sufficient to result in effects in SHSY5Y-htt-N63-148-Q cells similar to effects previously seen in HEK-293 cells. TG-catalyzed modifications of mutant huntingtin and cytotoxicity were attenuated in SHSY5Y-htt-N63-148Q cells expressing CaM-peptide compared with SHSY5Y-htt-N63-148Q cells expressing scram-CaM-peptide or GFP. These findings make it plausible that AAV-mediated expression of CaM-peptide could potentially have beneficial effects in an HD transgenic mouse model.

CaM-peptide was able to attenuate TG-catalyzed modifications to mutant huntingtin, but it was unclear whether this effect was specific to TG-catalyzed modifications of mutant huntingtin or if it was a non-specific effect on TG-catalyzed modifications of all TG substrates. We used two different approaches to measure total TG activity, an *ex vivo* approach in which total TG activity is measured in lysed cells, and an *in situ* approach in which an exogenous substrate is added to the media, taken up by cells and then modified by TG *in situ*. Using both the *ex vivo* and the *in situ* approach, TG activity was not significantly affected by expression of CaM-peptide in either SHSY5Y cells not expressing mutant huntingtin (non-htt-SHSY5Y) or in SHSY5Y-htt-N63-148Q cells. TG activity was significantly increased in SHSY5Y-htt-N63-148Q cells expressing scram-CaM-peptide or GFP compared with non-htt-SHSY5Y cells as measured with the *in situ* assay. This is similar to the disease state in which there is elevated TG activity in the brains of HD patients compared with control subjects (23, 25). However, TG activity in SHSY5Y-htt-N63-148Q cells expressing CaM-peptide was not significantly different from non-htt-SHSY5Y cells or from SHSY5Yhtt-N63-148Q cells expressing scram-CaM-peptide or GFP. The results suggest that CaM-peptide does not affect TG activity in cells without mutant huntingtin expression, further indicating that the effects of CaM-peptide are restricted to the site of interaction between TG and mutant huntingtin and therefore does not significantly affect total TG activity.

Based on the total TG activity assays, it appeared as though CaM-peptide specifically alters TG activity associated with mutant huntingtin but not total TG activity. However, it was unclear whether CaM-peptide also alters the activity of other CaM-dependent enzymes. This was important to investigate as not all CaM-dependent enzymes may have increased activity in HD like TG, or may not even be altered or involved in HD. TG protein levels and activity are elevated in human HD brains (24, 25) and there is increased TG activity as well as immunoreactivity in R6/2 HD transgenic mouse brains (11). In contrast to TG, CaM kinase II protein levels are decreased in R6/2 HD transgenic mouse brains (10), suggesting that there may be a subsequent decrease in CaM kinase II activity. However, we found that in SHSY5Y cells transfected with mutant huntingtin, there were no significant differences in CaM kinase II activity compared with control cells transfected with vector. This suggests that CaM kinase II activity may not be altered in HD as TG activity is. It is also important to determine whether expression of CaM-peptide would alter CaM kinase II activity. We found that expression of CaM-peptide did not significantly alter CaM kinase II activity in the HD-model cells or in control cells compared with cells not expressing CaM-peptide, suggesting that CaM-peptide does not alter the activity of other CaM-dependent enzymes. Collectively, these data suggest that CaM-peptide may specifically affect the CaM-mutant huntingtin interaction, thereby primarily altering TG-catalyzed modifications of mutant huntingtin.

As all of the previously observed effects of CaM-peptide have been in HD cell models, we used a cell-free *in vitro* assay to determine if there is a direct interaction between mutant huntingtin and CaM, and whether the CaM-peptide could interrupt the interaction. When purified recombinant huntingtin exon 1 with an expanded polyglutamine repeat was incubated with CaM-agarose, we were able to detect a direct interaction between the two proteins. This result differs from previous work in which an ^125^I-CaM overlay experiment was performed with purified rat huntingtin and ^125^I-CaM failed to bind huntingtin, suggesting that huntingtin and CaM did not bind directly (3). Differences in experimental design could account for the different outcomes. In comparison to the previous study that used full-length, wild-type huntingtin purified from rat brain, we used recombinant huntingtin consisting of exon 1 and having an expanded polyglutamine repeat containing 44 glutamines (htt-exon1-44Q). One possibility is that the polyglutamine expansion in the mutant form of huntingtin increases its affinity for CaM, allowing huntingtin to interact with CaM directly. Another possibility is that the exon 1 fragment of huntingtin has an altered conformation in which the CaM-binding region may be exposed, where otherwise it is masked in the full-length huntingtin structure. Furthermore, the wild-type huntingtin protein sample was resolved by SDS-PAGE and transferred to a membrane which was incubated with ^125^I-CaM. It has been shown that some proteins are unable to renature upon transfer to a membrane (4). Possibly, the CaM-binding domains of the 350 kDa huntingtin protein failed to renature on the membrane. We performed our CaM-binding experiments by incubating the htt-exon1-44Q protein sample with CaM-agarose, allowing huntingtin to be in a native state. Lastly, the relative protein concentrations used in the previous experiments may be problematic. In the previous ^125^I-CaM overlay experiments, ^125^I-CaM failed to bind purified rat huntingtin but bound the positive control, calcineurin. However, only 150 ng (0.43 pmoles) of purified rat huntingtin was used but 500 ng (6.25 pmoles) of calcineurin was used. Calcineurin is one of the highest affinity CaM-binding proteins examined to date (having a K_d_ = 28 pmols for binding of calcineurin to calcium-saturated CaM) (37). Therefore, it is likely that huntingtin has a lower affinity for CaM compared with calcineurin. In the previous study, an interaction between huntingtin and CaM may not have been detected simply because of the problem that there was not enough huntingtin present. In this study, 8.4 µg (560 pmoles) of recombinant htt-exon1-44Q was incubated with 45 µg (2.7 nmoles) of CaM which is within the range of amounts used in other protein-CaM-agarose binding studies: ∼37.5 µg (1.5 nmoles) of RGS4 with 26 µg (1.5 nmoles) of CaM (35); 10 µg (310 pmoles) of regucalcin with 90 µg (5.4 nmoles) of CaM (32); and 10 µg (500 pmoles) of FK506-binding protein (AtFKBP42) with CaM (22).

After a direct interaction between htt-exon1-44Q and CaM was established, we found that 10 µM of CaM-peptide was able to significantly inhibit the interaction between CaM and htt-exon1-44Q. The peptide concentration of 10 µM that was needed to inhibit CaM-mutant huntingtin binding is similar to concentrations required for other synthetic peptides to inhibit protein–protein interactions (14). The presence of W-5 did not affect the interaction between htt-exon1-44Q and CaM. These data suggest that in our previous study, the decreased TG-catalyzed modifications of huntingtin we observed when inhibiting CaM with W-5 (49) was most likely caused by inhibition of CaM activity and not caused by inhibiting the binding of CaM to mutant huntingtin. Furthermore, the presence of 664 µM W-5, but not lower concentrations (66 µM, 210 µM), attenuated the effect of 10 µM CaM-peptide, i.e. to inhibit CaM-mutant huntingtin binding. However, the amount of CaM-bound htt-exon1-44Q was still significantly less than when CaM-peptide was not present. This suggests that CaM-peptide may bind CaM at the site of interaction of W-5, but that CaM-peptide has a higher affinity necessitating a high concentration of W-5 (half a log more than the IC_50_ of 210 µM) (20, 40) to inhibit binding of CaM-peptide to CaM. Lastly, the lack of an effect of CaM-peptide on the interaction of CaM and calcineurin suggests that CaM-peptide may act directly on CaM and mutant huntingtin and not on other proteins that interact with CaM. As calcineurin has such a high affinity for CaM, the effect of CaM-peptide on the interactions of CaM and other CaM-binding proteins should be examined. However, the expression of the CaM-peptide reduced cytotoxicity in both the neuronal cell model and in a kidney cell model, which suggest that the CaM-peptide is not likely to interfere with crucial interactions of CaM with other proteins.

We previously demonstrated that CaM is associated with the aggregates of mutant huntingtin protein (49). Other lines of evidence also support a role for CaM in HD. The CaM binding protein, PEP-19, which inhibits calcium-CaM signaling (38, 39), has been shown to be decreased in HD brain regions that are vulnerable to degeneration (45). This decreased the inhibition of CaM, combined with the close proximity of TG and CaM (caused by the interaction of mutant huntingtin with both proteins) may contribute to CaM-mediated over-stimulation of TG. It has been shown that TG activity is increased in HD brains (23, 25). Increased TG activity can result in increased modifications and stabilization of aggregated and monomeric mutant huntingtin as well as oligomers of mutant huntingtin fragments. The binding of CaM to exon 1 of mutant huntingtin may result in the inhibition of the normal biochemical functions of CaM such as activating NOS. It has been shown that in R6/2 HD transgenic mice, NOS activity is reduced and NOS inhibition accelerates behavioral deficits (9). There are abnormally high levels of cytosolic calcium in HD transgenic mice (43), and mitochondria from HD patients display abnormal calcium homeostasis (33). Furthermore, huntingtin with an expanded glutamine repeat increases inositol 1,4,5-trisphosphate receptor-mediated calcium release (41–43). These deleterious effects on calcium homeostasis suggest that returning the functioning of CaM toward normal would be beneficial. The ability of the CaM peptide to inhibit the binding of CaM and N-terminal mutant huntingtin could result in increased functioning of CaM, assuaging the disturbed calcium homeostasis as previously shown in HEK-293 cells (13).

Our current findings illustrate that the expression of CaM-peptide in a neuronal HD cell model results in decreased TG-modifications of mutant huntingtin and decreased cytotoxicity. Our *in vitro* findings support the hypothesis that expression of a small fragment of CaM can regulate the binding of CaM and mutant huntingtin, and taken together with the cell culture data, suggest that interrupting this binding provides neuroprotective effects against the detrimental consequences associated with mutant huntingtin expression. Importantly, the CaM-peptide shows selective effects on TG-mediated modifications to mutant huntingtin and do not affect any other CaM-regulated enzymes or TG activity in general. The AAV-expressed CaM-peptide will next be tested in an HD transgenic mouse model. Inhibiting the interaction of CaM with mutant huntingtin protein is a potential target for therapeutic intervention in HD.
